# Senescent Remodeling of the Innate and Adaptive Immune System in the Elderly Men with Prostate Cancer

**DOI:** 10.1155/2014/478126

**Published:** 2014-03-19

**Authors:** Gianluigi Taverna, Mauro Seveso, Guido Giusti, Rodolfo Hurle, Pierpaolo Graziotti, Sanja Štifter, Maurizio Chiriva-Internati, Fabio Grizzi

**Affiliations:** ^1^Humanitas Clinical and Research Center, Via Manzoni 56, Rozzano, Milan 20089, Italy; ^2^Department of Pathology, School of Medicine, University of Rijeka, 51000 Rijeka, Croatia; ^3^Division of Oncology and Hematology, Texas Tech University Health Sciences Center, Lubbock, TX 79409, USA

## Abstract

Despite years of intensive investigation that has been made in understanding prostate cancer, it remains a major cause of death in men worldwide. Prostate cancer emerges from multiple alterations that induce changes in expression patterns of genes and proteins that function in networks controlling critical cellular events. Based on the exponential aging of the population and the increasing life expectancy in industrialized Western countries, prostate cancer in the elderly men is becoming a disease of increasing significance. Aging is a progressive degenerative process strictly integrated with inflammation. Several theories have been proposed that attempt to define the role of chronic inflammation in aging including redox stress, mitochondrial damage, immunosenescence, and epigenetic modifications. Here, we review the innate and adaptive immune systems and their senescent remodeling in elderly men with prostate cancer.

## 1. Introduction

Prostate cancer is the most prevalent malignancy in men worldwide and is a leading cause of cancer death [1, 2]. Several men with localized prostate cancer will never suffer any symptoms or adverse effects of the disease, but because of the difficulties in identifying this group of patients the majority receive radical local treatment, which can mainly result in erectile dysfunction and urinary leakage [3, 4]. The still open question for clinicians is deciding which men have “fast growing” cancers that need essential treatment and which men have “slow growing” cancers that will never trouble them [5]. Prognostic markers may help to avoid unnecessary treatment and identify patients with poor outcomes who would be candidates for trials of adjuvant treatment [6–9]. Based on the exponential aging of the population and the increasing life expectancy in industrialized Western countries, prostate cancer in elderly men is becoming a disease of increasing significance [10–12].

**Figure 1:**
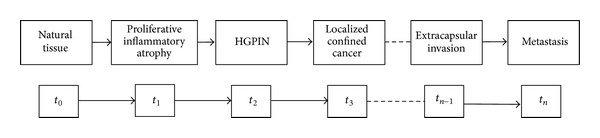
Multistate prostate carcinogenesis determined by the progression of different qualitative states identifiable in the development of cancer from normal tissue. The time parameter (*t*_0_, *t*_1_,…, *t*_*n*−1_, *t*_*n*_) depends on a large number of variables interconnected in many ways in a nonlinear manner. This makes it extremely difficult to predict the exact time interval between two successive states. Although carcinogenesis is a continuum, its differentiation into successive states is based on differences in histological and clinical data. Proliferative inflammatory atrophy (PIA) is a frequently observed lesion in prostate biopsies and some investigators have postulated its involvement in prostate carcinogenesis. PIA shares genetic alterations with high-grade prostatic intraepithelial neoplasia (HGPIN) and prostate cancer. HGPIN is currently regarded as the precursor lesion on the basis of pathological, epidemiological, and cytogenetic evidence. HGPIN lesions can be subdivided into at least four different architectural patterns.

It has been ascertained that the human prostate is the site of origin for the two most prevalent diseases of elderly men: benign prostatic hyperplasia (BPH) and prostate cancer [13, 14]. Prostate cancer is a highly heterogeneous disease encompassing a wide variety of pathological entities and a range of very different clinical behaviors [15]. This is underpinned at molecular level by a complex array of genetic alterations that affect cell processes, thus determining the dynamical progression of the neoplastic disease and its variable response to treatment (Figure 1) [16, 17]. Genomic alterations with a potential involvement in prostate cancer include somatic mutations, gene deletions or amplifications, and chromosomal rearrangements [17–21]. Epigenetic changes, more specifically DNA methylation, are the most common alterations in prostate cancer [22]. These changes are associated with transcriptional silencing of genes, leading to an altered cellular behavior. In light of this, epigenetic markers, especially the glutathione S-transferase pi gene (GSTP1), have been largely proposed as potential biomarkers for the evaluation of the probability of biochemical recurrence [23]. Other markers have a strong body of scientific data supporting their role in prostate cancer diagnosis, most notably adenomatous polyposis coli (APC), retinoic acid receptor beta (RARB), RAS association domain family protein 1 (RASSF1), CDH1, CDKN2A (p16), and the O(6)-methylguanine-DNA methyltransferase (MGMT) [22, 24]. Prostate cancer clinical phenotypes range from indolent or clinically insignificant to locally aggressive or metastatic [25–27]. A high number of gene expression profiling studies have been carried out to attempt the establishment of a “molecular” staging system, but the identification of genetic markers that predict aggressive disease has not yet been clinically demonstrated [28–33].

**Figure 2:**
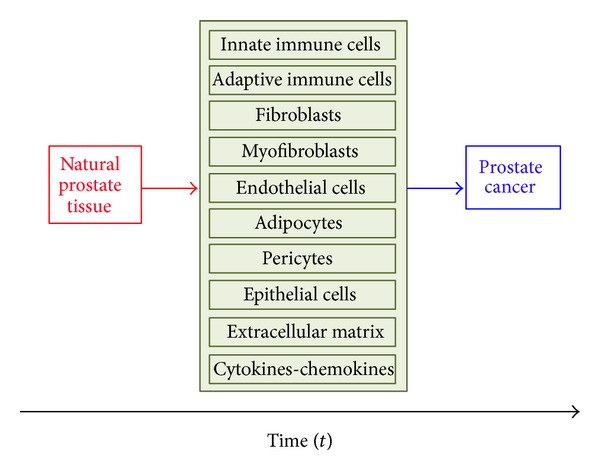
Histopathological examination reveals that prostate cancer is associated with diverse immune cell infiltrates and that, in the cancer context, epithelial cells coexist with extracellular matrix components and nonneoplastic cell types, including fibroblasts and endothelial cells, which collectively form the tumour stroma. Evidence supports the concept that tumour stromal cells are not merely a scaffold, but rather they influence growth, survival, and invasiveness of cancer cells, dynamically contributing to the tumour microenvironment. The interactions between epithelium and the surrounding stroma are required to maintain organ function and provide proliferative and migratory restraints that define anatomical and positional information, mediated by several growth factors, cytokines, chemokines, and extracellular matrix components. When cancer develops, transformed cells lose these constraints while stroma adapts and coevolves to support the “function” of the tumour.

Molecular associations with prostate cancer phenotypes continue to be fragmentary and, in some cases, have been poorly substantiated by follow-up investigations. Histopathological examination reveals that, like other solid tumors, prostate cancer is associated with diverse immune cell infiltrates and that, in the cancer context, epithelial cells coexist with extracellular matrix components and nonneoplastic cell types, including fibroblasts and endothelial cells, which collectively form the tumor stroma (Figure 2) [34–36]. Several lines of evidence support the concept that tumor stromal cells are not merely a scaffold, but rather they influence growth, survival, and invasiveness of cancer cells, dynamically contributing to the tumor microenvironment, together with immune cells [35, 37–40]. It is known that interactions between epithelium and the surrounding stroma are required to maintain organ function and that these interactions provide proliferative and migratory restraints that define anatomical and positional information, mediated by several growth factors and extracellular matrix components [41]. When cancer develops, transformed cells lose these constraints while stroma adapts and coevolves to support the “function” of the tumor [35]. The prostate represents an example of organ that relies on its surrounding stroma during normal development and cancer progression [35]. Jia et al. [42] compared Affymetrix gene expression profiles in stroma near tumor and identified a set of 115 probe sets for which the expression levels were significantly correlated with time-to-relapse. The authors compared patients that relapsed shortly after prostatectomy (<1 year) and patients that did not relapse in the first four years after prostatectomy and identified 131 differentially expressed microarray probe sets between these two categories. They conclude that tumor-adjacent prostate cancer stroma contains numerous changes in gene expression at the time of diagnosis that correlate with the chance of relapse following prostatectomy [42]. It is likely that the differences in RNA expression are often reflected in differences in chromatin modification, DNA methylation, and protein levels, which could also serve as stromal markers for progression [43]. Reinertsen et al. [44] showed that prostate fibroblast primary cultures from areas with cancer and hyperplasia with PC-3 cells seem to make the cancerous and hyperplastic fibroblasts more like each other, as the number of differentially expressed genes decreases.

The cells of the immune system that are commonly found infiltrating prostate cancer include IL^−^17^+^ macrophages [45, 46], neutrophils [47], mast cells [48], natural killer cells [49], and cells associated with an adaptive immune response, that is, T- and B-lymphocytes (Figure 2) [50–54]. Although it is commonly thought that an immune response localized to the tumor inhibits cancer growth, it is now clear that some types of tumor-associated inflammation may also exert an opposite action, at least at some point of prostate cancer natural history [55]. Here, we review the innate and adaptive immune systems and their senescent remodeling in elderly men with prostate cancer.

## 2. The Prostate Cancer in the Elderly

Prostate cancer, which is most often diagnosed in men over the age of 65 years, still remains one of the most common human cancers [11]. Treatment options vary depending on the stage and grade of the cancer, as well as patient comorbidity and age. More than one-half of men aged younger than 65 years are treated with radical prostatectomy [1]. Those aged 65 years to 74 years commonly undergo radiation therapy (nearly, 40%). Data show similar survival rates for patients with early stage disease who are treated with either of these methods. Active surveillance rather than immediate treatment is a commonly recommended approach, especially for older men and those with less aggressive tumors and/or more serious comorbid conditions [1, 56–59]. It is known that overdiagnosis and overtreatment are frequent in the elderly men. Competing mortality risks of men older than 75 years may supersede the risk of dying from prostate cancer several fold [10].

It is now accepted that aging is associated with an increase in a wide range of age-related diseases, including cardiovascular dysfunction, metabolic disorders, neurodegeneration, and cancer [60]. Even in the absence of identifiable disease, the physiology of organs, tissues, and cells declines throughout life. Within a tissue, both differentiated cells and adult stem cells are susceptible to intrinsic and extrinsic changes during aging [61–64]. In addition to these damages, cellular aging is also influenced by the exposure to extrinsic factors, including inflammatory cytokines [65–67]. It has been accepted that mammalian aging is associated with molecular, cellular, and physiological changes characterized by a deteriorating homeostatic balance associated with the increasing prevalence of neoplasia and other chronic diseases [68]. Although the correlation between inflammatory pathways and aging is now established, it remains difficult to demonstrate a causal connection [65]. Several theories have been proposed to define the role of chronic inflammation in aging. They include the redox stress, mitochondrial damage, immunosenescence, endocrinosenescence, epigenetic modifications, and age-related diseases. Immunosenescence, a state of gradual deterioration of the immune system brought on by natural aging, is felt to be a significant contributor to this increased risk. Careful analyses of healthy people ranging in age from neonates to centenarians suggest that a complex and continuous remodeling of the immune system occurs with age. In particular, deterioration of the immune system and the endocrine system during aging is thought to contribute to increased morbidity and mortality. It has been ascertained that bidirectional interrelations of both systems are present in the young and in the elderly; that is, endocrinosenescence modulates the immune system and immunosenescence changes the endocrine system. Terms such as alteration, deterioration, and decline do not account for the complexity of immunosenescence [69]. Hence, the more appropriate term of “senescent immune remodeling” has been proposed [70]. It is known that chronic inflammation might contribute to general aging in several ways. The perpetual presence of circulating proinflammatory factors may keep the immune system in a state of “chronic low-level activation.” This chronic immune activation might determine immunosenescence, caused primarily by an exhaustion of the pool of naïve T-cells, clonal expansion among T- and B-lymphocytes, and the consequent shrinkage of “immunological space”; together, these phenomena reduce the body's ability to respond to new antigens [71, 72]. In addition to causing immunosenescence, some proinflammatory factors (i.e., matrix metalloproteinase-3 (MMP-3)) may degrade the tissue microenvironment [73]. Several cytokines produced by senescent cells, including interleukin-6 (IL-6) and interleukin-8 (IL-8), are known as attractors and activators of innate immune cells, which can destroy tissue environments by virtue of the oxidizing molecules they release [74]. It is now ascertained that immunosenescence leads to increased incidence of infectious diseases morbidity and mortality as well as heightened rates of other immune disorders such as autoimmunity, cancer, and inflammatory conditions [75].

It is known that, after peak reproductive age, the histology of the prostate begins to undergo age-related changes that continue throughout life. Additionally, it has been shown that the size of the prostate typically increases throughout a man's lifetime and that different growth characteristics in each prostate zone may contribute to differences in the overall growth rate with age [76]. Turkbey et al. have recently shown that magnetic resonance imaging is able to document age-related changes in prostate zonal volumes [77]. Although key mechanisms are not yet completely understood, these changes might be attributed to altered androgen action and inflammatory processes that lead to either an unabated trophic effect on the gland and/or a chronic inflammation. It is possible that repeated epithelial insult sustained throughout the aging process results in a change of biology from one of differentiated reproductive function to one of chronic wound repair. Das et al. [78] have investigated aging-related changes in important cellular pathways, by systematically determining the effects of growth and development and aging on proteomic profiles in different lobes of the rat prostate. They found that proteins modulated during growth and development in the dorsolateral (DL) and ventral (VL) lobes are involved in a variety of biological processes including cell development, whereas proteins modulated during aging were predominantly related to antioxidant activity and immunity [78]. Interestingly, the importance of the Golgi apparatus in cellular activities as a stress sensor, apoptosis trigger, lipid/protein modifier, mitotic checkpoint, and a mediator of prostate malignant transformation has been highlighted [79]. Richie et al. [80] compared the levels of selenium, glutathione, and protein-bound glutathione (GSSP) in blood and prostate tissues in young (4 months), mature (12 months), old (18 months), and very old (24 months) male F344 rats. They found that, after 12 months, an 85% reduction in selenium in the DL was observed, while levels in other lobes were unchanged. In animals of all ages, levels of glutathione were the lowest in the VL than in the DL and no significant changes were observed in glutathione levels by 18 months. However, GSSP, a marker of oxidative stress, was increased 90% after 18 months in the DL only. These findings of age-related changes in GSSP and selenium in the DL prostate are consistent with the sensitivity of this lobe to carcinogenesis and, thus, may be playing a mechanistic role. It is known that androgens are involved in every aspect of prostate embryogenesis, and in aging men with prostatic hyperplasia. Likewise, androgen deprivation at any phase of life causes a decrease in prostate cell number and DNA content. The downstream control mechanisms by which hormonal signals are translated into differentiation, growth, and prostate function in elderly men are still unraveled [81, 82]. While many studies have explored the relationship between prostate cancer and serum androgens, the association remains ill-defined and clinical implications are difficult to recognize. Age remains one of the main factors that complicate the interaction between prostate malignancy and serum hormone levels, mainly as testosterone levels begin to decrease in the ageing man coincidentally as the incidence of prostate cancer starts to increase. Pierorazio et al. evaluated the relationship between testosterone levels and the development of high-risk prostate cancer and found that higher levels of serum free testosterone are associated with an increased risk of aggressive prostate cancer among older men [83]. They concluded that higher levels of serum free testosterone are associated with an increased risk of aggressive prostate cancer among older men and that appropriate clinical trials are compulsory to define the role of testosterone in the development of prostate cancer and insure the safety of testosterone-replacement therapy [83].

## 3. The Innate Immunity and Prostate Cancer

It has been widely demonstrated that the innate immune system is the first line of defense against infections. Although it is generally accepted that some aspects of innate immunity are well preserved in aging, several lines of evidence in the last decade support the notion that immunosenescence affects not only adaptive immunity but also innate immunity. Aside from T- and B-lymphocytes, innate immune cells orchestrate an inflammatory environment that may function to either stimulate or inhibit cancer growth. Various innate immunity cells have been implicated in prostate cancer onset, progression, and metastasis. Among these, macrophages are a primary source of secreted proinflammatory cytokines and are generally distinguished as type 1 (M1) or type 2 (M2) [84, 85]. M1s generally have an interleukin (IL) 12^low^IL-10^high^ phenotype, show impaired expression of reactive nitrogen intermediates and poor antigen presentation, and have tumoricidal capacity, while they show high expression of angiogenic factors (including vascular-endothelial growth factor (VEGF), epidermal-growth factor (EGF), and semaphorin 4D), MMPs, and cathepsins as well as of the growth arrest-specific protein GAS6 [86–88]. Additionally, M1 can support T-helper 1 (Th1) adaptive immunity [89]. Conversely, M2s secrete immunosuppressive cytokines and promote tumor growth [90]. It has been shown that cancer cells shape their interaction with macrophages by escaping phagocytosis and by promoting an M2-like polarization throughout chemokines and polarizing cytokines including chemokine ligand 2 (CCL2), colony stimulating factor 1 (CSF1), macrophage slowing factor (MSF), tumor necrosis factor-alpha (TNF-*α*), IL-10, and transforming growth factor-beta (TGF-*β*). Among the cells with M2 phenotype, the tumor-associated macrophages (TAMs) have been shown to be capable of secreting proteases that enhance invasion and metastases, together with a range of cytokines inhibiting an adaptive tumor-specific immune response, and angiogenic factors that increase neovascularity. It has been ascertained that phagocytosis is also unimpaired in the elderly [91]. Although macrophages are usually found located around necrotic areas of tumor and the advancing tumor margin, their role in prostate cancer still remains controversial. While it was originally thought that the main function of TAMs was direct cytotoxic effects on tumoral cells, phagocytosis apoptotic/necrotic cell debris, and present tumor-associated antigens to T-lymphocytes, current evidence suggests that inflammation and TAMs can also promote tumor growth and metastasis. The density, size, and location of tumor infiltrating macrophages in prostate cancer have been showed as powerful predictors of patient outcome, and prostate cancer specimens' harbor increased positive cells expressing the macrophage specific marker CD68 compared to benign glands [92–94]. Additionally, it was demonstrated that expression of macrophage colony-stimulating factor (M-CSF) and its receptor colony-stimulating factor-1 receptor (CSF-1R) are increased in primary tumors of patients exhibiting metastatic disease [95]. Shirotake et al. showed that both high monocyte chemoattractant protein-1 (MCP-1) expression and high macrophage infiltration in prostate cancer specimens correlate with a high prostate-specific antigen (PSA) recurrence rate and that AT1R blockade (ARB) inhibits MCP-1 expression through the PI3K/Akt pathway and blocks macrophage infiltration in castration-resistant prostate cancer [96]. Nonomura et al. [97] found that TAM infiltration was significantly correlated with serum PSA level, Gleason score, and clinical stage. Shimura et al. [98] demonstrated the association between TAM infiltration and disease-free survival after radical prostatectomy. They also examined the association between TAM infiltration and the rate of detection of prostate cancer at a repeat biopsy of the prostate in patients in whom the first biopsy was negative. They found no difference in TAM count between the cases with or without prostate cancer. By contrast, the macrophage scavenger receptor- (MSR-) positive inflammatory cells count in patients with cancer was significantly lower than that in patients without cancer at the repeat biopsy. Logistic regression analysis indicated that the MSR count at first biopsy is a significantly better predictive factor for positive repeat biopsy than PSA velocity, interval between first and repeat biopsies, or TAM count. Decreased infiltration of MSR-positive inflammatory cells in negative first biopsy specimens was correlated with positive findings in the repeat biopsy. MSR count has been, therefore, proposed as a valid index to avoid unnecessary repeat biopsies [97]. Gollapudi et al. [99] have recently shown that mean TAM number was higher in cancer cores versus prostatic intraepithelial neoplasia (PIN) and benign tissue and higher in high-grade prostate cancer supporting the potential role of TAMs in prostate cancer development. In a study comprising 38 prostate cancers, the presence of CD1a^+^ Langerhans cells was associated mainly with low-grade prostate carcinoma. Liu et al. [54] found a significant correlation between low numbers of CD1a^+^ cells and a high Gleason score and pathological stage pT3. The numbers of CD1a^+^ cells were, however, very low in normal and benign prostate tissues [54]. Other studies have, however, demonstrated variable evidence for TAMs during prostate cancer progression.

## 4. The Adaptive Immunity in Prostate Cancer

Ageing is accompanied by alterations to T-lymphocyte immunity and also by a low-grade chronic inflammatory state termed “inflammaging.” Immune cell infiltrate is a constant feature in normal prostate, BPH, and prostatic cancer [100]. It has been stated that the aged T-cell response is characterized by increased production of proinflammatory cytokines, which could significantly contribute to prostate carcinogenesis through induction of key inflammation-mediated prosurvival factors [101, 102]. Hussein et al. investigated the cells of the immune system present in normal prostate, BPH, and prostatic cancer [103]. It has been observed that PIA lesions are associated with chronic inflammation of the prostate, and histological transitions have been noted between areas of PIA and high-grade prostate intraepithelial neoplasia (HGPIN) and between PIA and prostate cancer [104]. The inflammatory infiltrates are mainly represented by CD3^+^ T-lymphocytes (70–80%, mostly CD4^+^ lymphocytes) and CD19^+^ or CD20^+^ B-lymphocytes (nearly, 10–15%) [105]. In the normal prostate, the infiltrates around the periglandular area are mainly composed of T-lymphocytes (70% CD8^+^ cells), while lymphoid aggregates are located in the fibromuscular stroma. These aggregates, mainly consisting of B-lymphocytes follicles surrounded by parafollicular T-lymphocytes with CD4^+^ cells, are two times more frequent than CD8^+^ cells. In the adult prostate, a different inflammatory infiltrate pattern has been described in relation to the type and extension of inflammation. Robert et al. [106] showed that, in 282 patients with BPH, there was an inflammatory infiltrate constituted by T-lymphocytes (i.e., CD3^+^ cells) in the 80% of cases associated with 52% of antigen-presenting cells, including B-lymphocytes (CD20^+^ cells). In contrast, a significant decrease in the counts of these cells was observed in high-grade prostatic cancer compared to BPH [105]. The increased density of CD3^+^ T-lymphocytes in BPH suggests that the initial response to cellular damage is mediated by cell-mediated immunity. The decreased density of immune cells in high-grade prostatic cancer may reflect immunosuppression. Recently, Fujii et al. found no significant difference in the number of infiltrating T-lymphocytes between benign and malignant tumors; however, the number of infiltrating B-lymphocytes was significantly reduced in malignant glands [107].

## 5. The Antigen-Presenting Machinery in Prostate Cancer

Defects in HLA class I antigen-processing machinery (APM) component expression often have a negative impact on the clinical course of tumors and on the response to T-cell-based immunotherapy [108]. Aged neutrophils are also less able to respond to rescue from apoptosis, while aged dendritic cells (DCs) are less able to stimulate T- and B-lymphocytes [109]. The altered T-lymphocyte stimulation is a result of changes in human leukocyte antigen expression and cytokine production, and lower B-lymphocyte stimulation is a result of changes in DCs immune complex binding [109]. It is known that DCs are the most potent, professional antigen-presenting cells of the immune system [110]. With their ability to interact with B- and T-lymphocytes and their widespread localization, they are a pivotal component of the innate immune system. The recently described positive outcome of a clinical trial with T-lymphocyte-based immunotherapy in prostate cancer has stimulated interest in the characterization of the APM component expression in prostate cancer lesions, since this “machinery” plays a crucial role in the generation and expression of the trimeric HLA class I surface antigen complex on tumor cells. Immunohistochemical staining of a tissue microarray including 59 primary prostate cancers lesions and matched normal tissues has shown downregulation of all the HLA class I APM components analyzed, although with a different frequency in tumor lesions when compared to normal prostate tissues [108]. A primary finding was the frequent loss or downregulation of calnexin and/or tapasin, which appears to be an independent prognostic marker of tumor recurrence. In contrast, HLA class I HC expression levels were less affected in primary prostate cancers; the frequency of downregulation described by Blades et al. has been found markedly lower than that reported by other authors [111] in a large number of prostate cancer lesions and that reported in other tumor types. In contrast, high frequencies of loss or downregulation of low molecular weight polypeptides 2 (LMP2), transporters associated with antigen processing 1 (TAP1), and tapasin as well as of *β*2-microglobulin were demonstrated in prostate cancer when compared to normal prostate tissue. The frequency of LMP2, TAP1, and tapasin downregulation is higher than that found in lung, colorectal, hepatocellular, cervical, and renal cell carcinoma. In parallel to the findings in head and neck squamous cell carcinoma, cervical carcinoma, esophageal carcinoma, breast carcinoma, and melanoma, the impaired TAP expression in prostate cancer lesions is associated with tumor grading, staging, and time to recurrence. Qiu et al. found that,* in vitro*, the prostate cancer cell PC-3 infected with Lentivirus TAP1 can efficiently overexpress TAP1 and tapasin, and HLA-1 was also upregulated on the surface of the infected cells. The Lentivirus TAP1 infection increased the apoptosis rate of PC-3 cells. In addition, with the coculture PC-3 cells and lymphocytes, TAP1 augmented the expression of CD3^+^ CD8^+^ CD38^+^ cells [112].

## 6. Immunotherapy and Prostate Cancer

The goals of any cancer therapy are to improve disease control, palliate pain, and overall survival [113]. In 2010, the American Food and Drug Administration (FDA) approved the first therapeutic cancer vaccine, called sipuleucel-T, for the treatment of castration refractory prostate cancer [114, 115]. Different from the currently adopted chemotherapy drugs that produce widespread cytotoxicity to kill tumor cells, anticancer vaccines and immunotherapies focus on empowering the immune system to overcome the tumor. It has been shown that prostate cancer is an ideal model for cancer vaccine development. This is mainly due to its humoral and cellular immunity to a range of cancer antigens, which are good candidates for vaccine therapy to generate a robust antitumor response. Recently, Cheema et al. suggested the potential application of BORIS (i.e., a cancer-testis antigen normally present at high levels in the testis and aberrantly expressed in various tumors and cancer cell lines) as a biomarker for prostate cancer diagnosis, an immunotherapy target, and, potentially, a prognostic marker of aggressive prostate cancer [116]. The ability of BORIS to activate the androgen receptor gene suggests its involvement in the growth and development of prostate cancer [116]. Chiriva-Internati et al. first reported the aberrant expression of the cancer-testis antigen A-kinase anchor protein-4 (AKAP-4) in prostate cancer, which will potentially be developed as a biomarker in prostate cancer. They also provide evidence that AKAP-4 is a potential target for prostate cancer adoptive immunotherapy or antitumor vaccination [117]. Beginning in the early 1990s, several tumor-associated antigen genes including the cancer-testis antigens were identified that exhibited tumor-specific expression. The cancer-testis antigens are a group of proteins that are typically restricted to the testis in the normal adult but are aberrantly expressed in cancers of unrelated histologic origin [118]. Hudolin et al. observed MAGE-A1 in 10.8% of carcinoma samples, whereas multi-MAGE-A and NY-ESO-1/LAGE-1 stained 85.9% and 84.8% of samples using immunohistochemistry, suggesting that a panel of CT antigens rather than individual ones may be more valuable as biomarkers [119]. Smith et al. suggested that multiple synovial sarcoma X chromosome breakpoint (SSX) proteins are expressed in metastatic prostate cancers, which are amenable to simultaneous targeting [120].

Enzalutamide (i.e., a second-generation androgen antagonist) has been recently approved for castration-resistant prostate cancer treatment. Ardiani et al. showed that enzalutamide mediated immunogenic modulation in TRAMP-C2 cells.* In vivo*, enzalutamide mediated reduced genitourinary tissue weight, enlargement of the thymus, and increased levels of T-cell excision circles. Because no changes were seen in T-lymphocytes function, as determined by CD4^+^ T-lymphocyte proliferation and Treg functional assays, enzalutamide was determined to be immune inactive [121]. The combination of enzalutamide and immunotherapy has been, therefore, suggested as a promising treatment strategy for castration-resistant prostate cancer. A renewed interest in prostatic acid phosphatase (i.e., a nonspecific phosphomonoesterase synthesized in prostate epithelial cells, whose level proportionally increases with prostate cancer progression) has been shown, because of its usefulness in prognosticating intermediate to high-risk prostate cancers and its success in the immunotherapy of prostate cancer [122]. Based on the good prognostic value of prostatic acid phosphatase and the potential usefulness of prostatic acid phosphatase as an antigen, an immunotherapy employing autologous prostatic acid phosphatase-loaded dendritic cells was initiated [123]. Wada et al. used a well-described genetically engineered mouse, autochronous prostate cancer model to explore the relative sequencing and dosing of anti-cytotoxic T-lymphocyte-associated antigen-4 (CTLA-4) antibody when combined with a cell-based, granulocyte macrophage colony-stimulating factor- (GM-CSF-) secreting vaccine [124]. These experiments corroborate recent clinical data, which suggest that the combination of CTLA-4 blockade and cell-based, GM-CSF-secreting vaccines may have significant antitumor effects in men with prostate cancer. These data also indicate that the “therapeutic window” of such an approach may be maximized through meticulous study of various dosing regimens. Additionally, future clinical studies may find that the addition of cyclophosphamide to this treatment strategy allows for reduction in the dose of anti-CTLA-4, potentially limiting autoimmune toxicity. In a recent phase I trial, Perez et al. demonstrated that the AE37 vaccine is safe and induces HER-2/neu-specific immunity in a heterogeneous population of HER-2/neu^+^ prostate cancer patients [125]. Clusterin is a cytoprotective chaperone protein that is overexpressed in many tumor types and is upregulated in response to cellular stress caused by cancer treatments, including hormonal manipulation, radiation, and chemotherapy. Custirsen is known as a second-generation antisense oligonucleotide that is complementary to clusterin mRNA and potently suppresses clusterin expression in preclinical models of prostate cancer as well as in clinical trials. The innovative first-in-human phase I neoadjuvant trial demonstrated dose-dependent plasma and prostate tissue concentrations of custirsen, which was well tolerated at all dose levels [126]. Results from the clinical trials (i.e., sipuleucel-T-based vaccine, GVAX-prostate cancer, viral prostate cancer vaccines, DNA-based vaccines, and gene-mediated cytotoxic immunotherapy) indicate that prostate cancer vaccines are generally safe and, encouragingly, capable of generating tumor-specific T-lymphocyte responses. It is becoming evident that prostate cancer patients with early-stage disease may be those who obtain the main benefits from vaccines.

## 7. Concluding Remarks

The primary risk for prostate cancer is aging, often associated with inflammation (Figure 2). Inflammation results in a tissue microenvironment that alters the normal prostate epithelial cell differentiation program and that accelerates the initiation of prostate cancer with a basal cell origin [127]. Despite the continuous progress, prostate cancer is one of the main cancers that affect men, especially older men. This is largely due to the fact that the tumoral mass cannot be identified using current imaging techniques. Prostate cancer can only be diagnosed on the basis of increased PSA levels associated with a low accuracy of the biopsy fragments and the well-known subjectivity of a pathologist's interpretation. This has led to many patients being overtreated, undertreated, or simply inappropriately treated and allowed the progression of the disease. Prostate cancer in the aging male will become an increasingly important and controversial health care issue. Because the median age of diagnosis for men with prostate cancer is greater than 75 years, prostate cancer can be considered a disease of the elderly. The aging population and lowering PSA threshold to 2.6 ng/mL will have the most significant impact on estimated new prostate cancer cases in 2021 [128]. Several disease-specific factors (i.e., stage, tumor grade, and PSA level) and patient-specific factors (i.e., age, comorbidity, and functional status) need to be considered in the decision-making process [129]. Rice et al. evaluated the outcomes between a variety of treatments for low-risk prostate cancer in patients of 70 years of age and older [130]. They found that patients managed on watchful waiting without secondary treatment had the poorest overall survival and that watchful waiting without secondary treatment represents a statistically significant predictor of overall mortality [130]. Specific to prostate cancer, it has been identified that macrophage interactions with tumor cells promote androgen resistance and increased prostate cancer invasion through tissue factor expression [12, 37].* In vivo* studies by Parrinello et al. showed a significant increase in infiltrating inflammatory cells including macrophages in the prostates of aged mice [38], reflecting the prominent role for immune cells during the aging process, which is linked to prostate cancer development. Identifying contributing factors in the tumor microenvironment, which modulate this cleavage event on tumor cells, is necessary for determining alternative therapeutic targets for a multimodality approach to inhibit the invasion steps of metastasis. Despite the technical advantages offered by robotic systems and other techniques, the diagnostic process requires further improvement. It is known that prostate cancer consists of distinct subpopulations of cancer cells, each with its own characteristic sensitivity to a given therapeutic agent. Cancer therapies can be seen as filters that remove the sensitive subpopulations but allow insensitive subpopulations to escape. The combined efforts of urologists, pathologists, gerontologists, and biologists can contribute much towards improving our understanding of the complexity of prostate cancer, and such a multidisciplinary approach will help to clarify existing concepts, categorize current knowledge, and suggest alternative approaches to the discovery of biomarkers and predictive values that urgently need to be translated into clinical practice.
